# Review and analysis of Chilean dental undergraduate education: curriculum composition and profiles of first year dental students

**DOI:** 10.1186/s12960-018-0314-8

**Published:** 2018-09-17

**Authors:** Renato E. Venturelli Garay, Richard G. Watt

**Affiliations:** Department of Epidemiology and Public Health, University College London, 1-19 Torrington Place, London, WC1E 6BT United Kingdom

**Keywords:** Chilean dental education, Dental students, Dental curriculum, Human resources for health, Dental professionals

## Abstract

**Background:**

In Chile, dentistry has become a very popular career choice for students, which has resulted in a substantial increase in both, the number of dental graduates and dental schools. Nonetheless, there is a need for change in the way dental schools select and educate their students to keep pace with the rapidly changing nature of societal needs and to tackle the marked health inequalities that exist in the country. The aim of this study was to review and critique dental undergraduate education in Chile, with a particular focus on the curriculum composition and profiles of students admitted to dental schools from 2010 to 2014.

**Methods:**

A descriptive and retrospective design was utilised. Two different methods were undertaken: primary data collection regarding curriculum and secondary data analysis in relation to students’ profiles. Descriptive statistics were used to assess the relative proportions of subject modules within the undergraduate dental curriculum and in particular the public health components. The analysis of the student profiles described specific background factors, namely, gender, age, secondary school type, location, rural-urban status and student’s year of admission. Also, trends of dental students’ intake between 2010 and 2014 were investigated. Logistic regression analysis was undertaken to assess potential associations between the aforementioned background factors and students’ choice of dental school.

**Results:**

Regarding the curriculum review, a 67% response rate was obtained. The most dominant component of Chilean dental curriculum was the clinical subjects (33%), followed by the basic and biological sciences (16%) and then medical and dental sciences (13%). In relation to the admission of students, the majority attended private schools (72%); most were females (62%); aged 19 years or less (74%); had an urban origin (99%); and came from subsidised private secondary schools (48%). Significant differences were found between students admitted to traditional and private dental schools.

**Conclusions:**

Clinical sciences are the most dominant subjects in the Chilean dental curriculum. Overall, traditional and private institutions had a broadly similar composition in their curriculum with the exception of the public health component. Students from disadvantaged backgrounds were the minority in dental schools across Chile.

## Background

Even though oral health is globally recognised as one of the major components for good health, the burden of oral disorders continues to remain high throughout the world, suggesting that dentistry has not been effective in preventing or controlling these diseases [1, 2]. Moreover, marked oral health inequalities persist both within and between countries [3, 4]. Oral health professionals should become strong advocates for a new vision for oral health [5]. Today’s dental training, oral healthcare delivery system and distribution of dental professionals are not focusing and adapting to the needs of most populations, creating a mismatch between the supply and demand of oral health professionals [6, 7]. Currently, many dental schools are not adequately integrated with the relevant local, regional and national health authorities to ensure a successful alignment between dental education, health delivery system and population oral health needs [8, 9].

A well-designed curriculum, which is responsive to the needs of the population, could contribute to improving the quality of services and to social accountability [10]. In addition, emphasising the public health focus of dental education, with a commitment to prepare dental students to become leaders in health promotion and disease prevention, has been highlighted [1, 11].

The growing diversity of patient population, the inequitable distribution and shortage of health professional in rural and urban-underserved areas has brought new attention to imbalances in the admission processes for health professionals [12]. The students that are usually admitted to health professions disproportionally come from higher social classes and dominant ethnic groups [12, 13].

In Chile, “traditional” universities refer to those formed before 1981, while “private” universities refer to those created after 1981 [14]. In recent years, Chilean dental schools have experienced an exponential growth, from five dental schools in 1997 to 34 faculties in 2011. Correspondingly, the number of students enrolled in dentistry has also increased from less than 2000 in 1997 to 12 000 in 2011 [15]. Students normally require to meet two conditions to access dentistry in Chile: firstly, to hold a Licencia de Educacion Media (awarded after completion of secondary school-level education) and, secondly, to have taken a national examination titled Prueba de Seleccion Universitaria (PSU) [16]. When students apply for entry to dental schools, they obtain a combined score based on their PSU results and their performance while in secondary school. Universities have a merit-based competition admission policy and thus, the level of entry varies between dental schools [17].

By the year 2025, a 77% oversupply of dentists is expected [15]. Nevertheless, inequalities in the utilisation and access of dental services remain as a matter of major concern for the Chilean government [18, 19]. This suggests that current models of dental workforce education and training might not be aligned with the diversity of patients they serve and with the population’s needs. Additionally, even though there is a national accreditation board, the Chilean universities have autonomy with regard to their curriculum content and educational methods [20] and as a result, no standard core-compulsory curriculum for dental schools exists [17].

In light of the recommendations proposed by several expert organisations worldwide [1, 10, 13], the purpose of this study was to investigate two aspects of dental education in Chile. The first objective was to describe and explore components of the Chilean dental curriculum, since there is a convincing movement to align the curriculum with population’s need as a mechanism to achieve the necessary competencies for a socially accountable practice [21]. The second objective was to assess the specific background characteristics of students admitted into different schools of dentistry in Chile to determine if any inequalities existed in the social backgrounds of students.

To the best of our knowledge, there is no published paper mapping out the overall current curriculum in Chilean dental schools, nor characterising the Chilean dental student at a national level and assessing their choice of dental school.

## Methods

This research was completed using two different methods: primary data collection of the Chilean dental schools’ curriculum and secondary data analysis of the Chilean dental students’ admission records.

### Study design and target population

A descriptive and retrospective design was undertaken. The target sample for the first part of the study consisted of all dental schools open in 2015 (33 faculties). For the second aspect, the target population were all first-year students enrolled into dentistry from 2010 to 2014. No sampling procedures were required as the study took the form of a census survey.

### Data collection

As to the curriculum review, a variety of sources were explored from March 2015 to July 2015. First, all dental schools’ websites were inspected in order to obtain each curriculum [22]. Second, e-mails were sent to all universities that did not provide online access to curriculum’s number of credits or hours per module. Then, a follow-up e-mail was sent when no reply was received. Finally, the lead researcher (RV) wrote to personal contacts to acquire further missing data. Data to address the second aspect of the study was collected by the Ministry of Education of Chile (MINEDUC). This institution produces and disseminates data regarding the Chilean educational system [23]. Therefore, it offers free access to anonymised databases under appropriate requests [24].

### Study variables

#### Curriculum review

Dental schools’ programmes were divided according to their content into nine different “groups of modules” based upon pragmatic educational considerations. The following categories were created and used to outline the Chilean curriculum structure:

- Basic and biological sciences
- Medical and dental sciences
- Humanities and social sciences
- Public health-related sciences
- Pre-clinical sciences
- Clinical management courses
- Clinical sciences
- Other courses
- Vocational training

#### Admission profiles

A database was created (based on MINEDUC data) that had a range of different variables, including student’s demographic, secondary school and dental school variables. The variable used as outcome was the type of dental school, categorised into two groups, the “traditional” universities and the “private” ones. The explanatory variables included were student’s age, gender, admission year, secondary school type, location and urban-rural status.

### Data analysis

With regard to curriculum review, only descriptive statistics were utilised. Mean numbers of modules taught and percentages for each “group of modules” were calculated based on the number of credits/hours in every curriculum. This was done with the aim of measuring the relative importance attributed to each “group of modules” by each dental school. Subsequently, all dental curriculum were clustered and compared according to whether they belonged to traditional or to private institutions. Regarding dental students’ admission, statistical analysis was performed on statistical software package STATA version 12.0 IC. *p* value of less than 0.05 was kept as the level for statistical significance for all tests. Missing data was explored and dropped in order to have the same number of observation in both descriptive and analytical parts of the analysis. Logistic regression was run in order to assess potential associations between specific students’ background factors and their choice of dental school type. Firstly, simple logistic regression models were run to assess the crude relationship between the dependent variable and each of the explanatory variables. Secondly, mutually adjusted logistic regression models were performed.

## Results

### Chilean dental curriculum

**Fig. 1:**
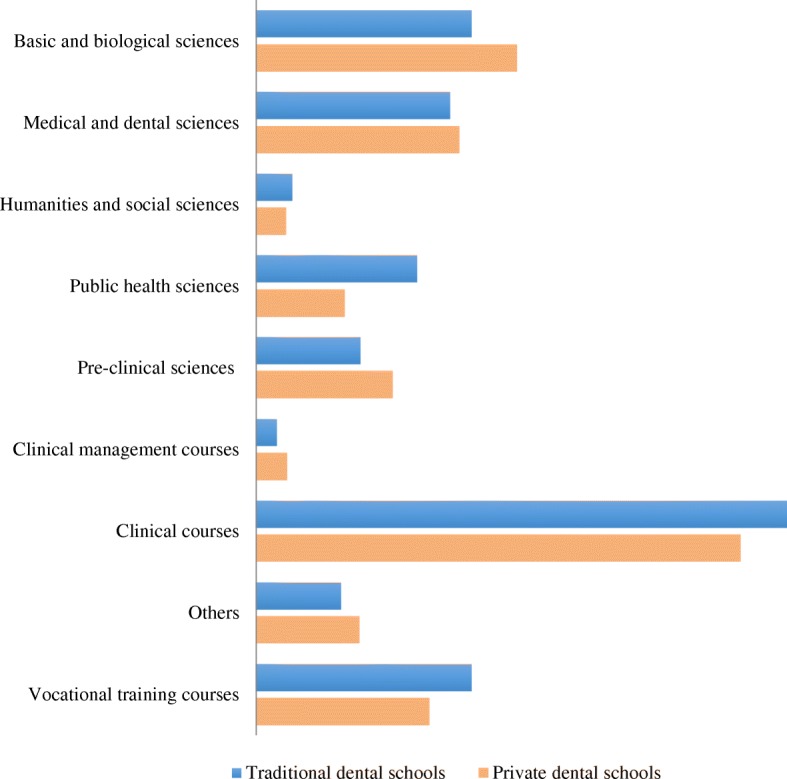
Relative proportion of “groups of modules” in traditional and private dental schools of Chile

**Fig. 2:**
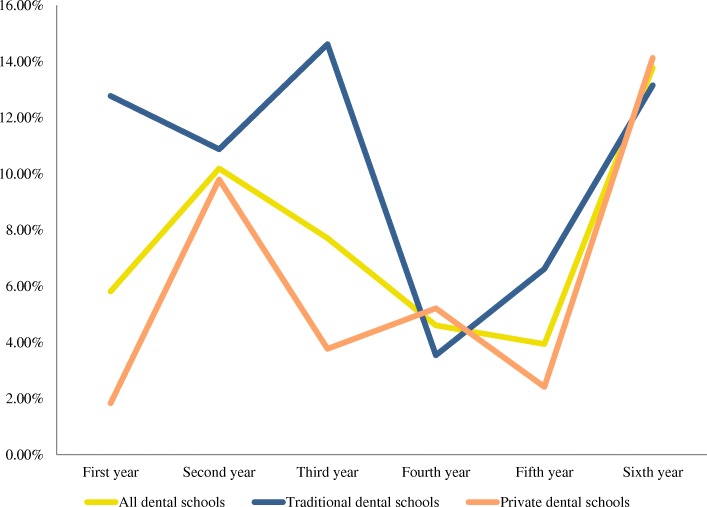
Distribution of dental public health sciences within Chilean dental curriculum

A total of 22 curriculum were obtained from dental schools across Chile, representing a response rate of 67%. Table 1 presents the results of the Chilean dental curriculum descriptive analysis. Both traditional and private dental schools attributed more importance to *clinical courses*, with 34% and 31% of their respective curriculum time devoted to it. Accordingly, the number of modules taught under this category represented the majority in the Chilean curriculum, with an average number of 12 modules by curriculum. It is apparent from Fig. 1 that this group dominates the Chilean dental curriculum. The *basic and biological sciences* occupied the second highest proportion of curriculum time with 16%, followed by the *medical and dental sciences* with 13%. It is noticeable that even though the *vocational training courses* showed a small mean number of modules (1.5), its percentage of curriculum time is similar to *medical and dental sciences*. *Public health sciences* represented 7% of the Chilean dental curriculum time. Nevertheless, differences were evident between dental schools’ types, with traditional dental schools attributing 10% of their curriculum to public health-related issues, while private dental schools attributing only 6%. Correspondingly, the mean number of public health-related subjects was higher among traditional dental schools’ curriculum (8.5) in comparison with private dental schools (4.4). In terms of the distribution of dental public health-related modules within the curriculum, Fig. 2 illustrates the scenario of all dental schools and for both, traditional and private institutions.

**Table 1:** Descriptive table of Chilean dental curriculum

Mean numbers and percentages of “categories of modules” in Chilean dental curriculum						
	Traditional universities		Private universities		All dental schools	
	Mean no. of modules	Relative proportion in curriculum time (%)	Mean no. of modules	Relative proportion in curriculum time (%)	Mean no. of modules	Relative proportion in curriculum time (%)
Basic and biological sciences	8.1	14	9.4	17	8.9	16
Medical and dental sciences	8.2	13	11	13	10	13
Humanities and social sciences	2.7	2	2.5	2	2.5	2
Public health sciences	8.5	10	4.4	6	5.9	7
Pre-clinical sciences	4.5	7	5.0	10	4.8	9
Clinical management	1.7	1	2	2	1.9	2
Clinical courses	15.5	34	10	31	12	33
Others modules	4.5	5	5.4	6	5.0	6
Vocational training courses	1.5	14	1.5	11	1.5	12

### Dental students’ admission

A total of 12 638 individuals out of 13 028 presented complete data. Table 2 provides the descriptive characteristics of the first year dental students. In terms of gender, 7.828 (62%) were females and 4 814 (38%) were males. The majority of Chilean dental students attended private dental schools (72%) rather than traditional dental schools (28%).

**Table 2:** Descriptive table of first year Chilean dental students and their distribution by dental school type

Characteristics	Traditional dental schools (*n* = 3 496–27.6%)	Private dental schools (*n* = 9 142–72.3%)	All dental schools (*n* = 12 638)
Gender			
Male	1 359 (38.87%)	3 455 (37.79%)	4 814 (38.09%)
Female	2 137 (61.12%)	5 687 (62.20%)	7 824 (61.91%)
Age			
≤ 19	2 943 (84.18%)	6 419 (70.21%)	9 362 (74.08%)
> 19	553 (15.82%)	2 723 (29.79%)	3 276 (25.92%)
Secondary school type			
Public school	663 (18.96%)	1 298 (14.20%)	1961 (15.52%)
Subsidised private school	1 722 (49.26%)	4 362 (47.71%)	6 084 (48.14%)
Private school	1 108 (31.69%)	3 442 (37.65%)	4 550 (36.00%)
Other	3 (0.09%)	40 (0.44%)	43 (0.34%)
Secondary school rural-urban status			
Urban	3 464 (99.08%)	9 084 (99.37%)	12 548 (99.29%)
Rural	32 (0.92%)	58 (0.63%)	90 (0.71%)
Secondary school location			
Central	2055 (58.78%)	6 755 (73.89%)	8 810 (69.71%)
North	672 (19.22%)	1 000 (10.94%)	1 672 (13.23%)
South	769 (22.00%)	1 387 (15.17%)	2 156 (17.06%)
Admission year			
2010	625 (17.88%)	1803 (19.72%)	2 428 (19.21%)
2011	637 (18.22%)	1930 (21.11%)	2 567 (22.43%)
2012	723 (20.68%)	2 112 (23.10%)	2 835 (30.31%)
2013	722 (20.65%)	1 703 (18.63%)	2 425 (19.19%)
2014	789 (22.57%)	1 594 (17.44%)	2 383 (18.86%)

In relation to the secondary school background, 15% of the individuals graduated from public schools, 48% from subsidised private schools, 36% from private schools and 0.3% from others types of secondary schools. Only 90 individuals came from schools located in rural areas, while an overwhelming 99% of students came from schools with urban backgrounds. Furthermore, 70% of the students graduated from schools located in the centre of Chile, while students from the north and south represented only 13% and 17% respectively.

**Fig. 3:**
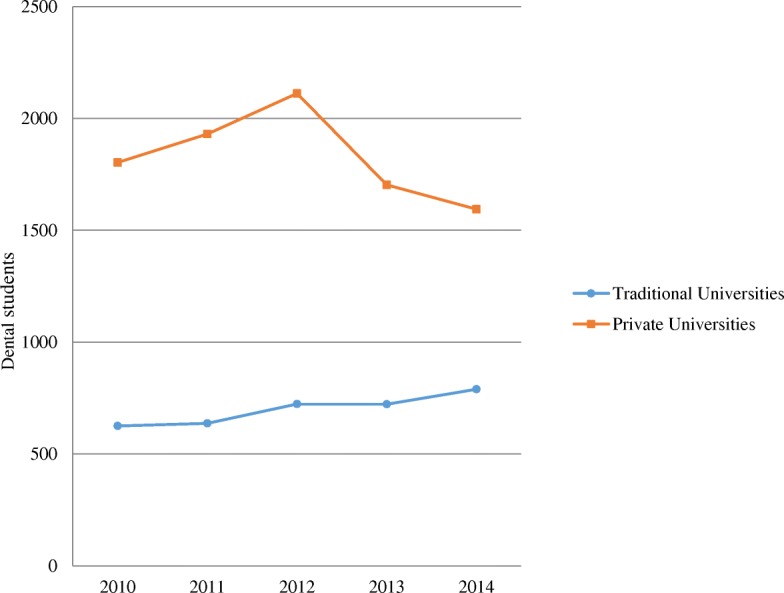
Traditional and private dental schools’ recruitment of dental students from 2010 to 2014

According to the admission year, the overall number of students admitted was steadily increasing from 2010 to 2012; however, in 2013, the intake of students started reducing (Fig. 3). Interestingly, the reduction occurred largely in private dental schools.

#### Association of private dental school enrolment and students’ specific background factors

Table 3 presents the results obtained from the analysis of the association between students’ background variables and private dental school admission. For the association of type of secondary school and private dental school admission, the results showed a clear gradient throughout the classification of type of secondary school. The odds of being enrolled into a private dental school increased by 1.44 times (95%CI 2.40 to 1.61) if students graduated from subsidised private schools, compared with students who graduated from public schools. Subsequently, the odds of studying in a private dental school became even higher (OR = 1.58, 95%CI 1.40 to 1.78) if students graduated from independent private schools. No association was found between gender and private school attendance (*p* > 0.05). In contrast, a strong association was observed when analysing the relationship between age and private dental school enrolment (*p* < 0,001). Students who are over 19 years of age were 2.29 (95%CI 2.07 to 2.55) times more likely to attend private dental schools compared to those in the younger age group. Likewise, a strong association was observed between private dental school attendance and student secondary school location. Students who graduated from secondary schools located in either the north or south of Chile were around 50% less likely to be enrolled in a private dental school than students who graduated from the central zone. The associations remained significant even after accounting for age, gender, secondary school type and year of admission (*p* < 0.001).

**Table 3:** Association between dental school type and secondary school type

Variables	Model 1^1^ OR (95% CI)	Model 2^2^ OR (95% CI)
Secondary school type		
Public school	1	1
Subsidised private school	1.29 (1.16–1.44)**	1.44 (1.28–1.61)**
Private school	1.58 (1.41–1.78)**	1.58 (1.40–1.78)**
Other	6.81 (2.09–22.09)**	5.60 (1.71–18.30)*
Gender		
Male	1	1
Female	1.04 (0.9–1.13)	1.04 (0.96–1.13)
Age		
≤ 19	1	1
> 19	2.25 (2.04–2.48)**	2.29 (2.07–2.55)**
Secondary school location		
Central	1	1
North	0.45 (0.40–0.50)**	0.44 (0.45–0.49)**
South	0.54 (0.49–0.60)**	0.55 (0.49–0.61)**
Admission year		
2010	1	1
2011	1.05 (0.92–1.19)	1.06 (0.93–1.21)
2012	1.01 (0.89–1.14)	1.01 (0.9–1.16)
2013	0.81 (0.72–0.92)*	0.87 (0.76–0.99)*
2014	0.70 (0.61–0.79)**	0.76 (0.67–0.87)**

^1^Unadjusted model

^2^Mutually adjusted model

*p < 0.05

**p < 0.001

Surprisingly, the associations between the years 2013 and 2014 with private dental school enrolment were significantly different in comparison with the year 2010 (*p* < 0.05; *p* < 0.001 respectively). Thus, students selected into dentistry in the years 2013 and 2014 were 0.87 (95%CI 0.76 to 0.99) and 0.76 (95%CI 0.67 to 0.87) times less likely to be recruited in private institutions than students selected in the year 2010. These associations remained significant after adjusting for age, gender, secondary school type and location.

## Discussion

### Composition of Chilean dental curriculum

The results of this study are very much aligned with what have been described in previous reports. For instance, the Institution of Medicine Report [25], the FDI vision 2020 [1] and the American Dental Education Association Commission on Change and Innovation [26] have all stated that dental education has been disproportionately focused on restorative care, overlooking public health-related issues. Likewise, one third of the entire curriculum time of Chilean dental schools was exclusively devoted to clinical courses and there was a lack of social sciences and public health subjects covered in their curriculum.

Today, there is a growing realisation that in order to obtain optimal oral health in society, a profound reorientation is needed to address the social determinants of oral health [27]. This is a powerful argument for strengthening the public health approach in dental education, with the vision to reshape and prepare dental professionals with a broader perspective towards oral health. Additionally, a strong understanding of health is needed to allow students to become advocates and leaders in oral health promotion and disease prevention [1]. The introduction of public health, political, social and psychological subjects in dental curriculum may be a step in the right direction.

### Students’ admission and enrolment

Students with disadvantaged background, namely, from lower socioeconomic positions, from remote and rural areas, are disproportionally fewer in higher educational institutions responsible for training health professionals. Chile is not the exemption as this study showed that students from more disadvantaged backgrounds are fewer in dental schools, regardless whether schools belonged to traditional universities or not. These results are coherent with what has been declared by the Lancet Commission [13], the WHO report on Transforming Health Workforce [10], the Global Consensus for Social Accountability of Medical Schools [28] and Rocha et al. (2012) [29] about the intake of health professionals.

All this suggests that inequalities in access to dental education are still present in the country. Correspondingly, the Chilean education system has been criticised as segmented and lacking in equity, where the quality and access to education differs according to the students’ socioeconomic position [30, 31].

Chilean dental schools have the responsibility of preparing dentists with the right combinations of skills, virtues and experiences to succeed in the workplace, but also to provide the right professional training to meet the needs of the communities they will serve. With the pronounced inequalities in access to oral healthcare in Chile, this is clearly not happening [19, 32]. Chile is a good example to show that oral health inequalities are not caused by the presence of too few dentists and that this major issue is not solved or improved by just educating more dentists. Improvements in equity of access to oral health services will not be reached without a better-balance distribution of oral health professionals between isolated and urban areas and between rich and poor regions of the country [13]. Without a serious commitment to expand the pool of students that reflects the country’s population, the access to oral healthcare will continue to be the same or worse. Policy-makers need to think deeply about the future of dental education and the role-played by the academic world on the dental workforce produced [33]. The dental educational system should be held responsible for meeting the needs of the entire Chilean population.

The most important condition in order to move forward is that Chilean dental education and dentistry in general must acknowledge that there is a crisis and dental education must embrace a new and appropriate pathway for the future.

### Strengths and limitations of the study

This is the first study that explored and described the overall composition of the Chilean dental curriculum. Likewise, it is the first study describing and assessing specific background characteristics of first year Chilean dental students regarding their choice of dental school. Both aspects of the study conducted census analysis, eliminating the bias induced by sampling design. However, this research did not analyse specific curriculum content, course goals and objectives or methods of teaching. Also, students’ academic results used to admit students into dentistry are possibly confounding the associations between their background factors and type of dental school attended. Individual socioeconomic position was not directly investigated; however data of secondary school type was used as a proxy.

### Recommendations and policy implications

In terms of curriculum design in Chile, it is recommended that dental schools revise their curriculum to better reflect the oral health needs of the population they serve and to reorient it towards oral health promotion and disease prevention, especially in private institutions. Additionally, to develop innovative models of education targeting to reduce oral health inequalities is a good step forward. Further research in the area is needed, in particular one making use of more comprehensive models of research as proposed by Bray and Thomas [34].

In relation with admission policies, it is important to increase the amount of underrepresented students in dental schools. Individuals from underrepresented backgrounds have a greater likelihood of serving their local communities [10]. Additionally, close monitoring and evaluation is needed to assess these processes. Academic institutions should take appropriate approaches to increase the diversity of the oral healthcare workforce, either diversifying their admission criteria of students or developing innovative models of education [35]. This could provide a platform to enhance the recruitment and retention of underrepresented students that could lead to tackling oral health inequalities.

## Conclusion

The most dominant subject components of the Chilean dental curriculum were the clinical courses. In general, traditional and private institutions showed similar curriculum composition. However, traditional schools placed greater emphasis on public health issues than private institutions.

Strong associations were found between age, secondary school type and location with attendance to private dental schools, demonstrating that significant differences exist between profiles of dental students enrolled in traditional universities and those attending private institutions.

## Abbreviations

FDI: World Dental Federation
MINEDUC: Ministry of Education, Chile
WHO: World Health Organization
